# Drug sensitivity patterns of HHV8 carrying body cavity lymphoma cell lines

**DOI:** 10.1186/1471-2407-11-441

**Published:** 2011-10-12

**Authors:** Rita Ötvös, Henriette Skribek, Lorand L Kis, Annunziata Gloghini, Laszlo Markasz, Emilie Flaberg, Staffan Eksborg, Jozsef Konya, Lajos Gergely, Antonino Carbone, Laszlo Szekely

**Affiliations:** 1 Department of Microbiology, Tumor and Cell Biology (MTC) and Center for Integrative Recognition in the Immune System (IRIS), Karolinska Institute, Box 280 SE-17177 Stockholm, Sweden; 2 Dipartimento di Anatomia Patologica, Istituto Nazionale Tumori, via Venezian, Milano, Italy; 3 Karolinska Pharmacy, and Department of Woman and Child Health, Childhood Cancer Research Unit, Karolinska Institutet, Karolinska University Hospital, Karolinksavagen, Stockholm, Sweden; 4 Department of Medical Microbiology, Medical and Health Science Center, University of Debrecen, Nagyerdei krt. 98, Debrecen, Hungary

## Abstract

**Background:**

Primary effusion lymphoma (PEL) is a rare KSHV/HHV8-associated high-grade non-Hodgkin's lymphoma (NHL) of B-cell origin, characterized by serous effusions in body cavities. Most patients are HIV-infected men with severe immunosuppression and other HHV8-associated diseases such as Kaposi's sarcoma (KS). The prognosis for those infected is poor, with a median survival of less than 6 months in most cohorts. Sustained complete remission is rare. High-dose chemotherapy regimens are used to improve remission rate and survival. The aim of the present study was to compare the drug sensitivity pattern of the available primary effusion (body cavity based) lymphoma-derived cell lines in order to find additional, potentially effective drugs that are not included in current chemotherapy treatment protocols.

**Methods:**

We have analyzed 11 cell lines against 27 frequently used cytostatic drugs in short term (3 days) survival assays using automated high throughput confocal microscopy.

**Results:**

All cell lines showed a distinct, individual drug sensitivity pattern. Considering the *in vitro *used and clinically achieved drug concentration, Vinorelbine, Paclitaxel, Epirubicin and Daunorubicin were the most effective drugs.

**Conclusions:**

We suggest that inclusion of the above drugs into PEL chemotherapy protocols may be justified. The heterogeneity in the drug response pattern however indicated that assay-guided individualized therapy might be required to optimize therapeutic response.

## Background

Human herpesvirus 8 (HHV8) or Kaposi sarcoma herpesvirus (KSHV) is the probable causative agent of two distinct lymphoproliferative disorders: primary effusion lymphoma (PEL) and the plasma cell variant of multicentric Castleman disease (MCD) in addition to Kaposi sarcoma (KS) [1].

Primary effusion lymphoma (PEL), or alternatively: body cavity lymphoma is a non-Hodgkin's lymphoma (NHL) of B-cell origin that develops predominantly in the serous body cavities [2]. The lymphoma cells, although lacking many conventional B-cell markers, carry immunoglobulin gene rearrangement and express syndecans, suggesting pre-plasma cell origin. At the clinico-pathological level, PEL is characterized by liquid growth in the serous body cavities associated with spreading along the serous membranes without infiltrative or destructive growth patterns [3,4]. Morphologically, PEL bridges immunoblastic and anaplastic features and frequently displays a certain degree of plasmacell differentiation. In all known cases, the monoclonal B-cell population is infected with HHV-8. Half of the lymphomas are dually infected with HHV-8 and Epstein-Barr virus (EBV) [5]. In the context of AIDS, most cases are associated with other KSHV/HHV8-related diseases such as Kaposi's sarcoma (KS) or multicentric Castleman's disease (MCD). As PEL typically lacks a solid component, its diagnosis rests on the cytological examination of body fluid. Phenotypically, expression of the CD45 antigen (> 90% of cases) confirms the lymphoid derivation of PEL cells, which exhibit an indeterminate immunophenotype, as they usually lack expression of B- and T-cell associated antigens (the majority of cases reported). There are, however, cases in the literature that had a B-cell or T-cell phenotype respectively [1].

Conversely, PEL cells generally express various markers associated with activation, including CD30 (approximately 75% of cases), CD38, CD71 and the epithelial membrane antigen. Moreover, PEL cells express several plasma cell markers including CD138, VS38c and MUM-1/IRF4 [1].

The prognosis of PEL is poor, as the median survival in the previously published series does not exceed 3 months [3,6-10].

Given its rarity, however, there are very few longitudinal observational series of patients with PEL and no large prospective trials have ever defined optimal treatment strategies [11].

Prior to the introduction of antiretroviral therapy, the therapeutic results were unsatisfactory in cohorts of HIV+ patients, despite the use of aggressive polychemotherapy regimens including anthracyclines. The significant improvement in the prognosis of AIDS-related lymphomas observed in the antiretroviral therapy era also applies to the PEL setting.

In addition, the routine use of growth factors, such as the granulocyte colony-stimulating factor (G-CSF), to avoid prolonged periods of neutropenia resulting from chemotherapy is standard practice for all AIDS-related lymphoma (ARL) patients.

Despite the improvement in therapeutical strategies during the last few years, there is no evidence of a cure for PEL patients with conventional systemic chemotherapy addressed to aggressive NHL. The suggested benefit of high-dose Methotrexate in association with CHOP (Cyclophosphamide, Doxorubicin, Prednisolone and Vincristine)-like regimens is negatively balanced by the hampered toxicity of Methotrexate in the presence of serous effusions [1].

Novel approaches for body cavity lymphoma therapy outside traditional chemotherapy have been suggested as well [11]. These include the addition of antiviral therapy as well as inhibition of specific cellular targets. Anti-tumor activity of the antiviral therapy directed against KSHV/HHV8 infection has been reported. This experience is based on single case reports. Patients with a diagnosis of PEL, related or not to HIV infection, experienced prolonged complete remission after the intracavitary administration of Cidofovir - an antiviral agent. Intracavitary Cidofovir, as well as interferon-α, may represent a reasonable choice in patients' refractory to conventional chemotherapy, or in elderly patients not eligible for more toxic systemic therapies [12].

Another approach may be to target NF-κB through the use of proteasome inhibition with drugs, such as Bortezomib that induces apoptosis of PEL cell lines *in vitro *[13].

In the present study we have investigated 11 different primary effusion (body cavity based) lymphoma-derived cell lines to compare the drug sensitivity pattern, in order to find new potentially successful chemotherapy agents, that are not used in current treatment protocols.

## Methods

### Cell lines and culture conditions

The following primary effusion (body cavity based) lymphoma-derived cell lines were used in the present study. CRO-AP/2, CRO-AP/5, CRO-AP/6, BC-2, BC-3 were established from pleural effusion, CRO-AP/3, HBL-6, BC-3, BCBL-1, JSC-1 were established from ascites fluid and BCP-1 from peripheral blood.

Body-cavity cell lines were cultured in IMDM (Sigma), supplemented with 20% heat-inactivated (at 56°C for 45 min) fetal calf serum (FCS, Sigma), 100 IU/ml penicillin (Sigma), 100 μg/ml streptomycin (Sigma) and 2 mM L-glutamine (Sigma). Cell lines were grown at 37°C in the presence of 5% CO_2_. Cultures were fed twice weekly with the above-mentioned medium; maintained at ca. 0.5 × 10^6 ^cells/ml. All cell lines were examined daily in their culture vessels under an inverted microscope. Absence of mycoplasma contamination was routinely assessed using staining with Hoechst 33258.

### *In vitro *drug sensitivity assay

*In vitro *drug resistance of body-cavity cell lines were assessed using a 3-day cell culture on microtiter plates. 27 drugs (Table 1) were tested, each at 4 different concentrations in triplicates on 384 well plates. Each well was loaded with 30 μl cell suspension containing 9000 cells. After three days of incubation the living and dead cells were differentially stained using fluorescent VitalDye (Biomarker Hungary). The precise number of living and dead cells was determined using a custom built-automated laser confocal fluorescent microscope (a modified Perkin-Elmer UltraView LCI) at the Karolinska Institute core Visualization Facility (KIVIF). The images were captured using the computer program QuantCapture 4.0 [14,15]. Image correction and counting of living and dead cells was carried out by the program QuantCount 5.0. All programs were created by the authors, using the symbol based graphical programming environment OpenLab Automaton (Improvision). The 15 control wells, that were used to determine the control cell survival (CCS), contained cells with only culture medium and 50 nl DMSO without drugs. 5 wells contained cells with culture medium alone. Comparing the two types of control wells no toxic effect of DMSO could be seen. Mean cell survival (MCS) was determined from the average of cell survival of all 11 body-cavity cell lines (Table 2).

**Table 1:** Chemotherapic agents used in the present study

			Clinical dose	Half time	*In vivo* AUC^72 hr^	*In vitro *used concentrations	*In vitro* AUC^72 hr^	Ref	QAUC
Antimetabolites	Folic acid	Methotrexate	12 g/m^2^	24	623,70	0,033 - 4,17	12,000-1499,976	[21]	0,019 - 2,405
	
	Purine	Cladribine	5 mg/m^2^	3	5,67	0,007 - 0,83	0,480-59,976	[22]	0,085-10,578
	
		Fludarabine	25 mg/m^2^	2	27,72	0,167 - 20,83	12,000-1499,976	[23]	0,433-54,112
	
		6-Mercaptopurin	85 mg/m^2^	4	138,60	0,556 - 69,44	39,997-4999,680	[24]	0,289-36,073
	
	Pyrimidine	Cytarabine	1 g/m^2^	2	221,76	0,133 - 16,66	9,596-1199,520	[25]	0,043-5,409
	
		Fluorouracil	400 mg/m^2^	0.25	69,30	0,333 - 41,66	23,996-2999,520	[26]	0,346-43,283
	
		Gemcitabine	1000 mg/m^2^	0.7	388,08	0,267 - 33,33	19,198-2399,760	[27]	0,049-6,184

Alkylating/alkylating-like	Nitrogen mustards	Chlorambucil	0.2 mg/m^2^	2	33,60	0,667 - 83,33	47,998-5999,760	[28]	1,429-178,564
	
	Platinum	Carboplatin	360 mg/m^2^	3	665,28	0,007 - 0,83	0,480-59,976	[29]	0,001-0,090
	
		Oxaliplatin	130 mg/m^2^	5.74	270,46	0,033 - 4,17	2,400-299,952	[30]	0,009-1,109

Spindle poison/mitotic inhibitor	Taxane	Docetaxel	85 mg/m^2^	0.6	24,95	0,067 - 8,33	4,798-599,760	[31]	0,192-24,040
	
		Paclitaxel	175 mg/m^2^	3	257,80	0,013 - 1,67	0,956-119,520	[27]	0,004-0,464
	
	Vinca	Vinblastin	1.7 mg/m^2^	0.83	1,71	0,00067 - 0,083	0,048-5,998	[32]	0,028-3,510
	
		Vincristine	1.32 mg/m^2^	2	1,55	0,00067 - 0,083	0,480-59,976	[33]	0,309-38,636
	
		Vinorelbine	80 mg/m^2^	40	498,96	0,007 - 0,83	4,798-599,760	[34]	0,010-1,202

Cytotoxic/antitumor antibiotics	Anthracyclin	Daunorubicin	1.5 mg/kg	18	531,56	0,033 - 4,17	2,400-299,952	[35]	0,005-0,564
	
		Doxorubicin	50 mg/m^2^	30	1091,48	0,013 - 1,66	0,956-119,520	[36]	0,001-0,110
	
		Epirubicin	90 mg/m^2^	15	604,21	0,013 - 1,66	0,960-119,952	[27]	0,002-0,199
	
	Streptomyces	Dactinomycin	1.5 mg/m^2^	36	8,98	0,003 - 0,42	0,240-29,995	[37]	0,027-3,340
	
		Bleomycin	8 IU/kg/day	6	33,26	0,008 - 1	0,576-72,000	[38]	0,017-2,165
	
		Mitomycin	20 mg/m^2^	1	4,44	0,003 - 0,33	0,317-39,600	[39]	0,071-8,929
	
		Hydroxyurea	15 mg/m^2^	2	630,00	0,333 - 41,66	23,996-2999,520	[40]	0,038-4,761

Topo-isomerase in-hibitors	Camptotheca	Topotecan	1.2 mg/m^2^	3	2,49	0,007 - 0,83	0,054-6,718	[41]	0,022-2,693
	
		Etoposide	100 mg/m^2^	4	221,76	0,133 - 16,66	9,596-1199,520	[25]	0,043-5,409
	

Other		Asparaginase	30000 IU/m^2^	8	26,56	0,033 - 4,17	2,400-299,952	[42]	0,090-11,294
	
		Bortezomib	1.45 mg/m^2^	40	21,62	0,007 - 0,83	0,480-59,976	[43]	0,022-2,774
	
		Prednisolone	1 mg/kg/day	3	41.58	0,16666 - 20,83	11,998-1499,760	[44]	0,289-36,069

**Table 2:** The average Mean Cell Survival (MCS) of the eleven body cavity lymphoma cell lines at different drug concentrations, expressed as the Q Area Under Curve values (QAUC).

		125 × dilution	25 × dilution	5 × dilution	1 × dilution		125 × dilution	25 × dilution	5 × dilution	1 × dilution
Effective drugs	**Chlorambucil**					**Epirubicin**				
	**MCS**	14.8560	46.6236	80.8708	84.7608	**MCS**	19.2643	37.3816	63.5034	85.1459
	**SD**	15.3967	12.9966	21.0326	22.0111	**SD**	14.6115	16.3379	19.2617	26.7694
	**QAUC**	1,4285	7,1426	35,7129	178,5643	**QAUC**	0,0016	0,0079	0,0397	0,1985
	**Paclitaxel**					**Dactinomycin**				
	**MCS**	17.0131	45.1537	67.7781	89.5709	**MCS**	7.9351	36.0115	67.0155	86.7944
	**SD**	24.0155	49.5533	35.9258	22.0081	**SD**	8.4436	31.4395	26.8592	24.4412
	**QAUC**	0,0037	0,0185	0,0927	0,4636	**QAUC**	0,0267	0,1336	0,6679	3,3397
	**Daunorubicin**					**Docetaxel**				
	**MCS**	20.7690	27.3619	63.7950	87.2387	**MCS**	19.7376	21.9731	37.6060	59.5325
	**SD**	25.0224	24.1810	31.5421	23.0699	**SD**	21.0333	20.3415	21.1706	27.0225
	**QAUC**	0,0045	0,0226	0,1129	0,5643	**QAUC**	0,1923	0,9616	4,8081	24,0404
	**Vinorelbine**					**Vinblastin**				
	**MCS**	18.1346	20.5045	40.0830	53.0230	**MCS**	26.8433	40.9968	67.9391	81.0989
	**SD**	16.8776	21.5082	22.6266	26.5102	**SD**	32.9835	31.9132	33.5183	17.2183
	**QAUC**	0,0096	0,0481	0,2404	1,2020	**QAUC**	0,0281	0,1404	0,7020	3,5100
	**Asparaginase**					**Fluorouracil**				
	**MCS**	33.4151	62.3275	90.6113	94.4347	**MCS**	41.1156	58.9125	70.3122	83.0342
	**SD**	25.9953	34.9567	26.1363	23.3606	**SD**	19.1314	27.2339	23.4010	19.8986
	**QAUC**	0,0903	0,4517	2,2587	11,2937	**QAUC**	0,3463	1,7313	8,6566	43,2831
	**Etoposide**					**Doxorubicin**				
	**MCS**	30.6138	50.2125	64.2707	74.6943	**MCS**	29.0927	66.8651	77.2076	80.7702
	**SD**	20.8709	23.8410	23.6330	19.0535	**SD**	19.1960	25.0111	26.0959	27.3229
	**QAUC**	0,0433	0,2164	1,0818	5,4091	**QAUC**	0,0009	0,0044	0,0219	0,1095
	**Gemcitabin**					**Methotrexate**				
	**MCS**	31.4369	45.9785	58.4073	67.9991	**MCS**	47.1542	44.9205	70.0761	84.2659
	**SD**	33.0357	38.5124	37.0529	36.6568	**SD**	22.4628	21.6895	25.4751	25.4385
	**QAUC**	0,0495	0,2473	1,2367	6,1837	**QAUC**	0,0192	0,0962	0,4810	2,4050
	**Vincristine**					**Topotecan**				
	**MCS**	43.8400	69.1932	80.2086	81.6665	**MCS**	41.8048	75.3319	84.1050	90.7907
	**SD**	34.7208	35.8971	29.9816	32.6203	**SD**	16.4675	24.5366	25.2812	25.3696
	**QAUC**	0,3091	1,5455	7,7273	38,6364	**QAUC**	0,0215	0,1077	0,5385	2,6926

Non-effective drugs	**Bortezomib**					**Bleomycin**				
	**MCS**	79.4105	66.8656	68.2865	64.8357	**MCS**	60.3729	69.3422	76.5090	80.8431
	**SD**	25.4732	25.6668	28.0206	24.9547	**SD**	16.7323	18.8710	22.7197	16.8950
	**QAUC**	0,0222	0,1110	0,5548	2,7739	**QAUC**	0,0173	0,0866	0,4330	2,1650
	**Cladribine**					**6-mercaptopurin**				
	**MCS**	81.0033	82.0998	84.1061	92.0852	**MCS**	74.0197	87.8677	97.1334	98.2332
	**SD**	21.2666	22.0174	20.1630	21.3224	**SD**	28.3769	23.3376	30.0858	25.3860
	**QAUC**	0,0846	0,4231	2,1156	10,5778	**QAUC**	0,2886	1,4429	7,2146	36,0729
	**Oxaliplatin**					**Cytarabine**				
	**MCS**	87.0156	89.3148	82.6868	86.8853	**MCS**	69.9787	77.2199	83.5057	89.0542
	**SD**	30.3307	26.7413	24.1939	25.6311	**SD**	31.2151	36.2648	38.6377	32.5155
	**QAUC**	0,0089	0,0444	0,2218	1,1090	**QAUC**	0,0433	0,2164	1,0818	5,4091
	**Prednisolone**					**Mitomycin**				
	**MCS**	98.9838	99.8113	95.6985	92.9011	**MCS**	73.4043	79.2375	80.0565	79.4133
	**SD**	12.7979	15.5728	14.2013	14.0330	**SD**	28.5533	19.7620	18.7334	19.3133
	**QAUC**	0,2886	1,4428	7,2139	36,0693	**QAUC**	0,0714	0,3571	1,7857	8,9286
	**Hydroxyurea**					**Carboplatin**				
	**MCS**	88.8788	86.3322	97.5959	98.8578	**MCS**	113.3868	103.2988	113.4341	110.1786
	**SD**	21.5473	19.8568	17.5610	18.7218	**SD**	39.2983	27.4344	43.5238	38.6427
	**QAUC**	0,0381	0,1904	0,9522	4,7611	**QAUC**	0,0007	0,0036	0,0180	0,0902
	**Fludarabine**									
	**MCS**	67.0129	76.1851	79.2040	95.8649					
	**SD**	43.0343	35.3340	37.6906	19.6811					
	**QAUC**	0,4329	2,1645	10,8223	54,1117					

### Drugs

For the *in vitro *drug sensitivity test 27 drugs were used (summarized in Table 1). All the drugs were dissolved in 50% dimethyl sulfoxide (DMSO) - 50% phosphate buffered saline (PBS) and were printed on the 384 well plates using high-density array replicator metal pins with 50 nl replica volumes in a Biomek 2000 fluid dispenser robot (Beckman). The same robot was used to generate the drug masterplates containing the triplicates of four different drug dilutions (1 ×, 5 ×, 25 ×, 125 ×) using a single tip automatic pipettor dispenser head. The starting concentration of the dilution series for the individual drugs was initially determined based on the solubility of the different agents.

The drug plates that were used in this study were also tested on a large number of *in vitro *tumor cell lines and cells from primary tumor samples. In these assays we could show that it was possible to find sensitive cell lines for each individual drug, demonstrating that all the drugs on the plate were active [16,17] (data not shown).

To calculate the relationship between the *in vitro *drug concentrations and the *in vivo *ones, we used **area under curve **(AUC; area under the plasma, concentration curve versus time) values of the individual drugs. For this comparison Quotient of Area Under Curve values (QAUC^72 hr^) were determined by the following formula:

$$in\;vitro\text{ used concentration}\times \text{72 hours(}\mu \text{g}\times \text{hr}∕\text{ml)}∕in\;vivo\text{ AU}{\text{C}}^{\text{72hr}}\left(\mu \text{g}\times \text{h}∕\text{ml}\right)$$

The *in vivo *AUC^72 hr ^corresponds to the area under curve value achieved in patients under a 72 hours period. The *in vivo *AUC^72 hr ^was established from the clinical dose and half-time using the standard trapezoidal rule calculation. The *in vivo *AUC^72 hr ^data is summarized in the seventh column of Table 1. The detailed references to the clinical dose and to the *in vivo *halftime data are available at the Swedish pharmacological website http://fass.se. A QAUC^72 hr ^value higher than 1 indicates that the *in vitro *drug concentration is higher than the one used in the clinical practice. If this value is 1, it means that the *in vitro *concentration corresponds to the clinically achieved *in vivo *concentration.

## Results

### The *in vitro *drug sensitivity assay

**Figure 1:**
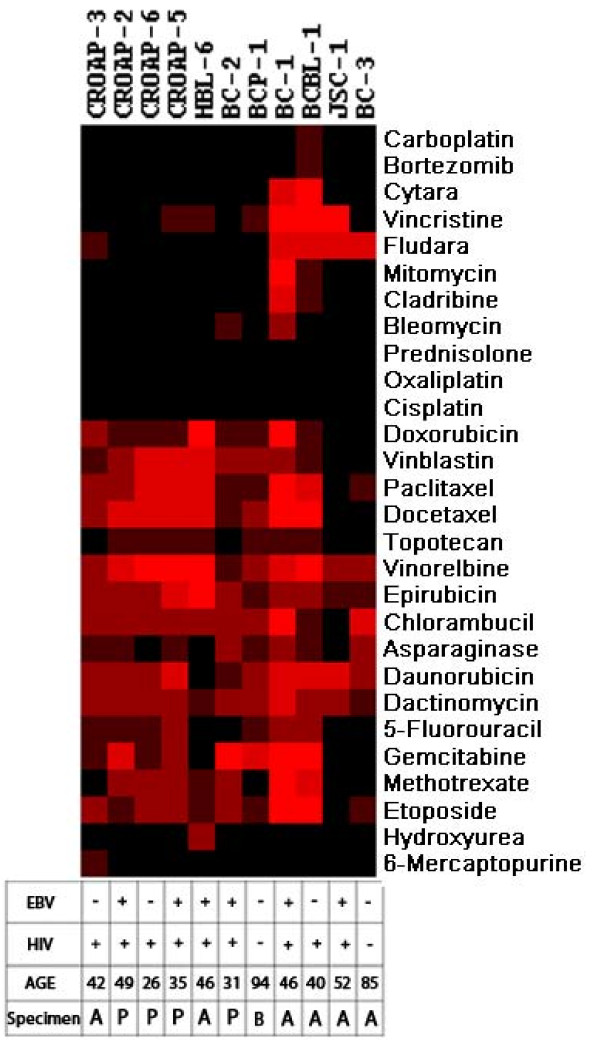
**Heat map representation of the hierarchical clustering of the simplified drug sensitivity data of the individual cell lines against all the drugs along with the presence of EBV in the cell lines, the HIV status and the age of the patients and the anatomical location of the founder sample (A - ascites, P - pleural effusion, B - blood)**. Intensity of the color shows the scale of the sensitivity. Black is resistant, brightness of the red color is proportional to the effectiveness of the drug.

**Figure 2:**
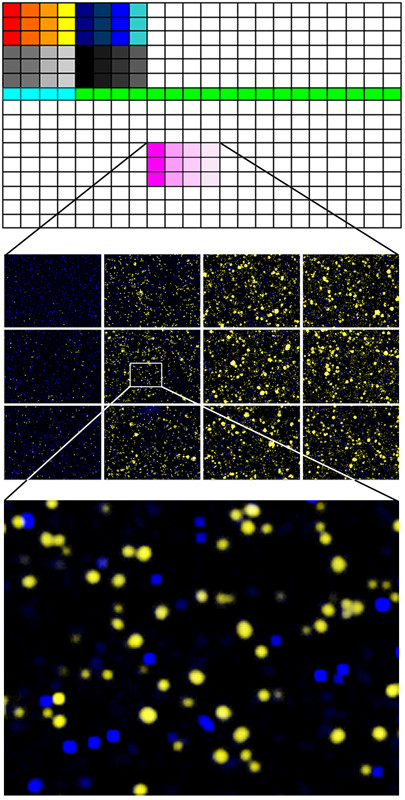
***In vitro *drug resistance of body cavity cell lines was assessed using a 3 day survival assay on microtiter plates on single cell level**. The top panel shows the 384 well plates, the colored area shows the drugs, each at 4 different concentrations in triplicates. The highest concentrations are on the left and each adjacent column represents a 5-fold serial dilution. The middle panel is the magnification of a mosaic of microscopic images of six wells treated with the dilution series of one drug. The living and dead cells were differentially stained using fluorescent dyes as shown in yellow and blue colors on the digitally colored images. The bottom panel shows a close-up of a single microscopic field within one well.

We have tested the drug sensitivity patterns of the body cavity lymphoma lines in short term, *in vitro *survival assays. The clinical origin and viral status of the individual lines is summarized in Figure 1. Each cell line was tested against 27 different drugs, in triplicates, at four different concentrations. The assay was carried out on 384 well plates. After 3 days of incubation each individual well of the test plates was photographed using a custom developed, automated extended field confocal microscope. Living and dead cells were differentially stained using viability dependent fluorescent dyes as shown in Figure 2. Each individual living or dead cell was identified counted and their fluorescence intensity distribution was recorded using automated image analytic and quantitation programs. For each well the percentage of surviving cells was calculated by comparing the number of living cells in the given well to the average of living cells in the untreated control wells.

### The summarized drug sensitivity pattern of the body cavity lymphoma cell lines

**Figure 3:**
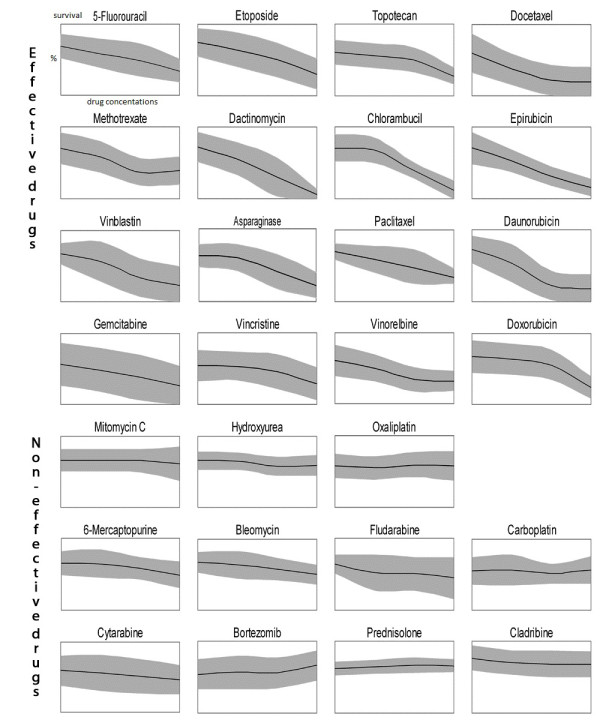
**Mean values and standard deviations of drug sensitivity of eleven body cavity cell lines for 27 different cytostatic drugs; x axis: increasing concentrations of the drug, y axis: fraction of surviving cells 0-100%**. The drug was considered to be effective if the survival was below 50% at least at one concentration. Non-effective drugs: survival is above 50% even at the highest concentration.

The summarized cell survival data is shown in Figure 3. The middle line of the individual curves represents the Mean Cell Survival (MCS) for all the cell lines along with the ± Standard Deviations of the means (SD - gray shaded area) for the four different dilutions of the 27 drugs. Drugs were considered to be more universally active if they showed less standard deviation around the means.

Most of the lines were sensitive for sixteen of the 27 drugs where sensitivity was defined as less than 50% mean survival at any of the drug dilutions (effective drugs). If more than half of the cells were alive even at the highest concentration than the drug was considered to be ineffective.

We found that the sixteen effective drugs against body-cavity lymphoma were the following in the order of effectiveness: Dactinomycin, Chlorambucil, Paclitaxel, Vinorelbine, Epirubicin, Docetaxel, Daunorubicin, Vinblastin, Doxorubicin, Etoposide, Gemcitabine, Asparaginase, Fluorouracil, Topotecan, Vincristine and Methotrexate.

Most body-cavity lymphoma lines were resistant to Oxaliplatin, Bleomycin, 6-Mercaptopurine, Hydroxyurea, Cladribine, Carboplatin, Bortezomib, Cytosine-arabinosid, Prednisolone, Mitomycin and Fludarabin. Although the Oxaliplatin, Cisplatin and Prednisolone drugs were not effective against any of the body-cavity lymphoma lines these drugs show concentration-dependent growth-inhibitory effect on other cell lines or primary tumors in parallel experiments (data not shown) at the same concentration as used in this paper [18].

### Heat map of the cluster analysis

In order to identify possible co-segregation of the sensitivity patterns of the individual drugs as well as to systematically compare all the lines with each other, we have carried out unsupervised two-dimensional hierarchical clustering of the simplified drug sensitivity data using the Cluster 3.0 program for MacOS X. The results were visualized using the program TreeView [19]. The sensitivity to the drug was represented on a 5 step scale where every step represents less than 50% viability at the four different drug dilutions. (Resistant - if more than 50% survival at the highest concentration, maximum sensitivity - if less than 50% survival at the lowest concentration.) The graphical representation of the clustering results, along with the EBV, HIV status and the presence of concomitant Kaposi sarcoma, are shown in Figure 1.

### Pharmacokinetic comparison

**Figure 4:**
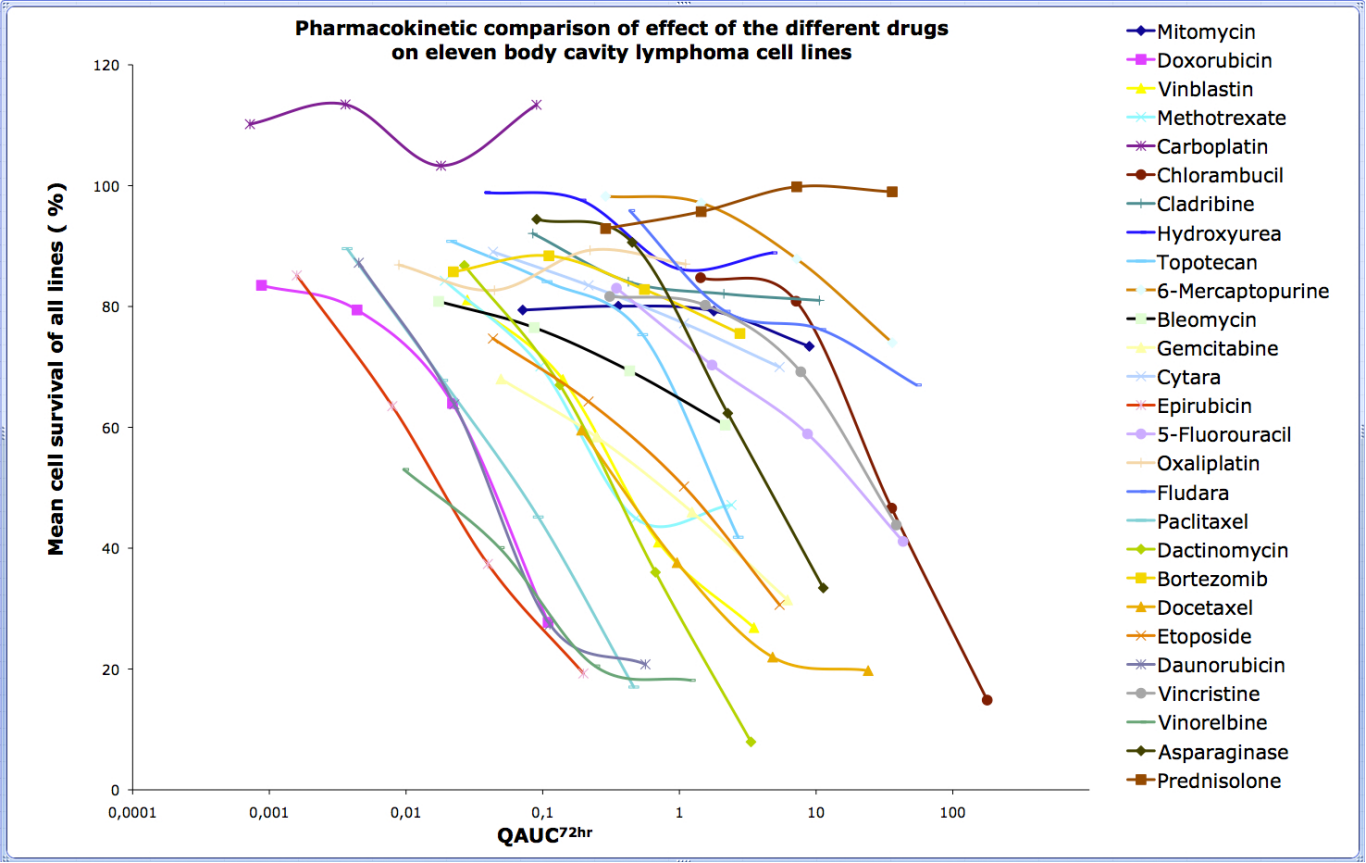
**Drug sensivity mean values of the survival of the body cavity lymphoma lines, plotted against QAUC^72 hrs ^values identify Epirubicin, Daunorubicin, Vinorelbine and Paclitaxel as novel candidate drugs against body cavity lymphomas**.

Absolute drug sensitivity values have relatively little clinical relevance if they are not correlated with clinically achievable *in vivo *concentrations. In order to analyze the data in relation to the pharmacokinetic behavior of the given drugs we have plotted the mean survival values as the function of the Quotient of the Area Under Curve (QAUC^72 hr^) values of the particular drugs. The QAUC^72 hr ^values of a drug were created by dividing the calculated *in vitro *AUC^72 hr ^values by the *in vivo *achievable AUC values (which were calculated from clinical dose and half-time). As shown in Figure 4. most drugs were tested in the pharmacologically most relevant range of QAUC^72 hr ^(close to 1).

Plotting the mean cell survival for each individual drug against a common QAUC^72 hr ^axis shows that Daunorubicin, Epirubicin, Paclitaxel and Vinorelbine were the most effective drugs (low survival at low QAUC^72 hr ^values). Moreover most body cavity lymphoma lines were sensitive to these drugs. Importantly, Doxorubicin, the only antracyclin drug that is currently included in chemotherapy protocols against body cavity lymphomas showed a rather heterogeneous effect. Two of the eleven lines were highly sensitive for Doxorubicin whereas two were completely resistant at the maximum drug concentration that we could reach in the current assay (QAUC = 0.11). The two lines (JSC-1 and BC-3) were, in general, the least sensitive for chemotherapeutic drugs, however were still sensitive for Daunorubicin, Epirubicin and Vinorelbine.

Dactinomycin showed the highest killing efficiency in the present *in vitro *assay. The calculation of the QAUC^72 hr ^value for the corresponding Dactinomycin concentration however revealed that the concentration that was required for the high killing effect is higher than the levels that are realistically achievable in a patient.

When treating the body-cavity lymphoma cells with Carboplatin at low QAUC^72 hr ^values, a relative increase in the number of surviving cells was observed as compared to non-treated controls. The survival was above 100% in case of all the 11 lines suggesting that low dose Carboplatin protected from spontaneous cell death.

## Discussion

*In vitro *growing cell lines are the closest model systems available today for studying the biological features of body cavity lymphomas. The cell lines that were used in the present study represent a variety of different origin. The investigation included cell lines established from ascites fluid, pleural effusion or from the peripheral blood of PEL patients. Despite their different origin, the body-cavity lymphoma lines showed a remarkably similar sensitivity pattern for a number of drugs. Only one cell line was highly resistant for most of the drugs (JSC1) whereas two cell lines (BC1 and BCBL-1) showed increased overall sensitivity to most of the drugs.

The presented data suggests that, for a number of cytostatic drugs the body cavity lymphoma cell lines share a common cytotoxic drug sensitivity profile. These profiles show no obvious correlation with the biological or clinical features of the lymphomas. Clustering of the drug sensitivity data revealed that the profiles are independent of the EBV status, anatomical localization of the lesion, the age the patient or the rapidity of the progression of the disease. The two cell lines (BCP-1 and BC-3) that arouse from HIV negative patients showed relatively low drug sensitivity.

The current treatment alternative is a combination of Methotrexate with CHOP (Cyclophosphamide, Doxorubicin, Prednisolone, Vincristine)-regimes. The present data showed that the cell lines exhibit varying sensitivity to Methotrexate and Vincristine and are completely resistant to Prednisolone.

Cyclophosphamide or Ifosphamide were not tested on the body cavity lymphoma lines, because both of these compounds are prodrugs that have to be converted into active metabolites by the liver *in vivo*.

It has been reported that the proteasome inhibitor Bortezomib induces apoptosis of the cell lines BCBL-1 and BCP-1 *in vitro *[13]. In this study only BCBL-1 showed detectable sensitivity to Bortezomib and only at the highest concentration whereas all the other lines were resistant.

In the present study body cavity lymphoma lines showed considerable sensitivity for anti-microtubule drugs and anthracyclins. Importantly all lines were sensitive to Epirubicin and Vinorelbine even at low QAUC^72 hr ^values. Epirubicin required tenfold lower concentration than the *in vivo *achievable concentration to kill more than 80% of the cells for most of the lines. Epirubicin is primarily used against breast and ovarian cancer, gastric cancer, lung cancer and lymphomas, but has not yet been tested against body cavity lymphomas [20].

In summary, the analysis of drug sensitivity profiles of the available body cavity lymphoma lines against 27 commonly used drugs revealed considerable heterogeneity in drug response. Four drugs, namely, Daunorubicin, Epirubicin, Paclitaxel and Vinorelbine showed uniformly high efficiency on the cell lines. These drugs are not yet included in the current chemotherapy protocols of body cavity lymphomas. The heterogeneity of drug response also suggests that optimal care of the lymphoma patients would include the determination of drug sensitivity patterns of the primary tumor samples and that these patients would benefit from assay guided individualized therapy.

## Conclusions

We suggest that inclusion of the above drugs into PEL chemotherapy protocols may be justified. The heterogeneity in the drug response pattern however indicated that assay guided individualized therapy might be required to optimize therapeutic response.

## Competing interests

The authors declare that they have no competing interests.

## Authors' contributions

The project was conceived and designed by LS. The experiments were mainly carried out and/or coordinated by ÖR. LLK, AG and LM took part in cell culturing, and preparation of the microtiter plates for *in vitro *drug sensitivity assays. EF was responsible for measuring the plates using the automated laser confocal fluorescent microscope. LS and EF wrote the computer programs QuantCapture 4.0 and QuantCount 5.0. LM and HS analysed and interpreted the data. SE made comparable the *in vitro *results with the *in vivo *data. JK, LG, AC together with the other authors have been involved in the planning of the experimental details, and the drafting and critical reading of the manuscript. All authors read and approved the final manuscript.

## Pre-publication history

The pre-publication history for this paper can be accessed here:

http://www.biomedcentral.com/1471-2407/11/441/prepub

## References

[B1] Carbone A Gloghini A KSHV/HHV8-associated lymphomas Br J Haematol 2008 140 1 13 24 10.1111/j.1365-2141.2007.06879.x 17991301

[B2] Boulanger E Daniel MT Agbalika F Oksenhendler E Combined chemotherapy including high-dose methotrexate in KSHV/HHV8-associated primary effusion lymphoma Am J Hematol 2003 73 3 143 148 10.1002/ajh.10341 12827649

[B3] Komanduri KV Luce JA McGrath MS Herndier BG Ng VL The natural history and molecular heterogeneity of HIV-associated primary malignant lymphomatous effusions J Acquir Immune Defic Syndr Hum Retrovirol 1996 13 3 215 226 10.1097/00042560-199611010-00003 8898666

[B4] Morassut S Vaccher E Balestreri L Gloghini A Gaidano G Volpe R Tirelli U Carbone A HIV-associated human herpesvirus 8-positive primary lymphomatous effusions: radiologic findings in six patients Radiology 1997 205 2 459 463 10.1148/radiology.205.2.9356629 9356629

[B5] Nador RG Cesarman E Chadburn A Dawson DB Ansari MQ Sald J Knowles DM Primary effusion lymphoma: a distinct clinicopathologic entity associated with the Kaposi's sarcoma-associated herpes virus Blood 1996 88 2 645 656 8695812

[B6] Ansari MQ Dawson DB Nador R Rutherford C Schneider NR Latimer MJ Picker L Knowles DM McKenna RW Primary body cavity-based AIDS-related lymphomas Am J Clin Pathol 1996 105 2 221 229 10.1093/ajcp/105.2.221 8607449

[B7] Otsuki T Kumar S Ensoli B Kingma DW Yano T Stetler-Stevenson M Jaffe ES Raffeld M Detection of HHV-8/KSHV DNA sequences in AIDS-associated extranodal lymphoid malignancies Leukemia 1996 10 8 1358 1362 8709643

[B8] Karcher DS Alkan S Human herpesvirus-8-associated body cavity-based lymphoma in human immunodeficiency virus-infected patients: a unique B-cell neoplasm Hum Pathol 1997 28 7 801 808 10.1016/s0046-8177(97)90153-2 9224748

[B9] Valencia ME Martinez P Moreno V Laguna F Lahoz JG AIDS-related body cavity-based lymphomas, herpesvirus-8 and HIV infection: a study of seven cases Aids 1999 13 18 2603 2605 10.1097/00002030-199912240-00021 10630536

[B10] Boulanger E Agbalika F Maarek O Daniel MT Grollet L Molina JM Sigaux F Oksenhendler E A clinical, molecular and cytogenetic study of 12 cases of human herpesvirus 8 associated primary effusion lymphoma in HIV-infected patients Hematol J 2001 2 3 172 179 10.1038/sj.thj.6200096 11920242

[B11] Chen YB Rahemtullah A Hochberg E Primary effusion lymphoma Oncologist 2007 12 5 569 576 10.1634/theoncologist.12-5-569 17522245

[B12] Halfdanarson TR Markovic SN Kalokhe U Luppi M A non-chemotherapy treatment of a primary effusion lymphoma: durable remission after intracavitary cidofovir in HIV negative PEL refractory to chemotherapy Ann Oncol 2006 17 12 1849 1850 10.1093/annonc/mdl139 16766593

[B13] An J Sun Y Fisher M Rettig MB Antitumor effects of bortezomib (PS-341) on primary effusion lymphomas Leukemia 2004 18 10 1699 1704 10.1038/sj.leu.2403460 15343345

[B14] Flaberg E Stuber G Szekely L Multi-dimensional laser confocal microscopy on live cells in submicroliter volumes using glass capillaries Acta Histochem Cytochem 2006 39 4 103 106 10.1267/ahc.05056 PMC1698864 17327896

[B15] Flaberg E Sabelstrom P Strandh C Szekely L Extended Field Laser Confocal Microscopy (EFLCM): combining automated Gigapixel image capture with in silico virtual microscopy BMC Med Imaging 2008 8 13 10.1186/1471-2342-8-13 PMC2515298 18627634

[B16] Markasz L Kis LL Stuber G Flaberg E Otvos R Eksborg S Skribek H Olah E Szekely L Hodgkin-lymphoma-derived cells show high sensitivity to dactinomycin and paclitaxel Leuk Lymphoma 2007 48 9 1835 1845 10.1080/10428190701559132 17786721

[B17] Markasz L Stuber G Flaberg E Jernberg AG Eksborg S Olah E Skribek H Szekely L Cytotoxic drug sensitivity of Epstein-Barr virus transformed lymphoblastoid B-cells BMC Cancer 2006 6 265 10.1186/1471-2407-6-265 PMC1664586 17101045

[B18] Skribek H Otvos R Flaberg E Nagy N Markasz L Eksborg S Masszi T Kozma A Adam E Miseta A Chronic lymphoid leukemia cells are highly sensitive to the combination of prednisolone and daunorubicin, but much less to doxorubicin or epirubicin Exp Hematol 2010 38 12 1219 1230 10.1016/j.exphem.2010.09.001 20837094

[B19] de Hoon MJ Imoto S Nolan J Miyano S Open source clustering software Bioinformatics 2004 20 9 1453 1454 10.1093/bioinformatics/bth078 14871861

[B20] Cersosimo RJ Hong WK Epirubicin: a review of the pharmacology, clinical activity, and adverse effects of an adriamycin analogue J Clin Oncol 1986 4 3 425 439 10.1200/JCO.1986.4.3.425 3005521

[B21] Crews KR Liu T Rodriguez-Galindo C Tan M Meyer WH Panetta JC Link MP Daw NC High-dose methotrexate pharmacokinetics and outcome of children and young adults with osteosarcoma Cancer 2004 100 8 1724 1733 10.1002/cncr.20152 15073863

[B22] Albertioni F Lindemalm S Reichelova V Pettersson B Eriksson S Juliusson G Liliemark J Pharmacokinetics of cladribine in plasma and its 5'-monophosphate and 5'-triphosphate in leukemic cells of patients with chronic lymphocytic leukemia Clin Cancer Res 1998 4 3 653 658 9533533

[B23] Hersh MR Kuhn JG Phillips JL Clark G Ludden TM Von Hoff DD Pharmacokinetic study of fludarabine phosphate (NSC 312887) Cancer Chemother Pharmacol 1986 17 3 277 280 10.1007/BF00256699 2427240

[B24] Chan GL Erdmann GR Gruber SA Stock P Chen S Ascher NL Canafax DM Pharmacokinetics of 6-thiouric acid and 6-mercaptopurine in renal allograft recipients after oral administration of azathioprine Eur J Clin Pharmacol 1989 36 3 265 271 10.1007/BF00558158 2526020

[B25] Gruber A Liliemark E Tidefelt U Paul C Bjorkholm M Peterson C Liliemark J Pharmacokinetics of mitoxantrone, etoposide and cytosine arabinoside in leukemic cells during treatment of acute myelogenous leukemia--relationship to treatment outcome and bone marrow toxicity Leuk Res 1995 19 10 757 761 10.1016/0145-2126(95)00061-r 7500654

[B26] Casale F Canaparo R Serpe L Muntoni E Pepa CD Costa M Mairone L Zara GP Fornari G Eandi M Plasma concentrations of 5-fluorouracil and its metabolites in colon cancer patients Pharmacol Res 2004 50 2 173 179 10.1016/j.phrs.2004.01.006 15177306

[B27] Fogli S Danesi R Gennari A Donati S Conte PF Del Tacca M Gemcitabine, epirubicin and paclitaxel: pharmacokinetic and pharmacodynamic interactions in advanced breast cancer Ann Oncol 2002 13 6 919 927 10.1093/annonc/mdf164 12123338

[B28] GlaxoSmithKline Research Triangle Park N Prescribing Information Leukeran^®^ http://www.drugs.com/monograph/leukeran.html

[B29] Ghazal-Aswad S Calvert AH Newell DR A single-sample assay for the estimation of the area under the free carboplatin plasma concentration versus time curve Cancer Chemother Pharmacol 1996 37 5 429 434 10.1007/s002800050408 8599865

[B30] Graham MA Lockwood GF Greenslade D Brienza S Bayssas M Gamelin E Clinical pharmacokinetics of oxaliplatin: a critical review Clin Cancer Res 2000 6 4 1205 1218 10778943

[B31] Rischin D Ackland SP Smith J Garg MB Clarke S Millward MJ Toner GC Zalcberg J Phase I and pharmacokinetic study of docetaxel in combination with epirubicin and cyclophosphamide in advanced cancer: dose escalation possible with granulocyte colony-stimulating factor, but not with prophylactic antibiotics Ann Oncol 2002 13 11 1810 1818 10.1093/annonc/mdf305 12419756

[B32] Bates SE Bakke S Kang M Robey RW Zhai S Thambi P Chen CC Patil S Smith T Steinberg SM A phase I/II study of infusional vinblastine with the P-glycoprotein antagonist valspodar (PSC 833) in renal cell carcinoma Clin Cancer Res 2004 10 14 4724 4733 10.1158/1078-0432.CCR-0829-03 15269145

[B33] Desai ZR Van den Berg HW Bridges JM Shanks RG Can severe vincristine neurotoxicity be prevented? Cancer Chemother Pharmacol 1982 8 2 211 214 10.1007/BF00255486 6125275

[B34] Freyer G Delozier T Lichinister M Gedouin D Bougnoux P His P Imadalou K Trillet-Lenoir V Phase II study of oral vinorelbine in first-line advanced breast cancer chemotherapy J Clin Oncol 2003 21 1 35 40 10.1200/jco.2003.09.057 12506167

[B35] Andersson B Andersson I Beran M Ehrsson H Eksborg S Liquid chromatographic monitoring of daunorubicin and daunorubicinol in plasma from leukemic patients treated with daunorubicin or the daunorubicin-DNA complex Cancer Chemother Pharmacol 1979 2 1 15 17 10.1007/BF00253099 498413

[B36] Toffoli G Corona G Cattarossi G Boiocchi M Di Gennaro G Tirelli U Vaccher E Effect of highly active antiretroviral therapy (HAART) on pharmacokinetics and pharmacodynamics of doxorubicin in patients with HIV-associated non-Hodgkin's lymphoma Ann Oncol 2004 15 12 1805 1809 10.1093/annonc/mdh464 15550586

[B37] Veal GJ Cole M Errington J Parry A Hale J Pearson AD Howe K Chisholm JC Beane C Brennan B Pharmacokinetics of dactinomycin in a pediatric patient population: a United Kingdom Children's Cancer Study Group Study Clin Cancer Res 2005 11 16 5893 5899 10.1158/1078-0432.CCR-04-2546 16115931

[B38] Peng YM Alberts DS Chen HS Mason N Moon TE Antitumour activity and plasma kinetics of bleomycin by continuous and intermittent administration Br J Cancer 1980 41 4 644 647 10.1038/bjc.1980.110 PMC2010280 6155927

[B39] Kozuch P Hoff PM Hess K Adams J Newman RA Lee F Pazdur R Phase I bioequivalency study of MitoExtra and mitomycin C in patients with solid tumors Cancer 2001 91 4 815 821 11241251

[B40] Yan JH Ataga K Kaul S Olson JS Grasela DM Gothelf S Kutlar A Orringer E The influence of renal function on hydroxyurea pharmacokinetics in adults with sickle cell disease J Clin Pharmacol 2005 45 4 434 445 10.1177/0091270004273526 15778424

[B41] Gerrits CJ Schellens JH Burris H Eckardt JR Planting AS van der Burg ME Rodriguez GI Loos WJ van Beurden V Hudson I A comparison of clinical pharmacodynamics of different administration schedules of oral topotecan (Hycamtin) Clin Cancer Res 1999 5 1 69 75 9918204

[B42] Ylikangas P Mononen I Serious neutropenia in ALL patients treated with L-asparaginase may be avoided by therapeutic monitoring of the enzyme activity in the circulation Ther Drug Monit 2002 24 4 502 506 10.1097/00007691-200208000-00007 12142634

[B43] Papandreou CN Daliani DD Nix D Yang H Madden T Wang X Pien CS Millikan RE Tu SM Pagliaro L Phase I trial of the proteasome inhibitor bortezomib in patients with advanced solid tumors with observations in androgen-independent prostate cancer J Clin Oncol 2004 22 11 2108 2121 10.1200/JCO.2004.02.106 15169797

[B44] Penzak SR Formentini E Alfaro RM Long M Natarajan V Kovacs J Prednisolone pharmacokinetics in the presence and absence of ritonavir after oral prednisone administration to healthy volunteers J Acquir Immune Defic Syndr 2005 40 5 573 580 10.1097/01.qai.0000187444.38461.70 16284534

